# Retracted: miR-509-5p Inhibits the Proliferation and Invasion of Osteosarcoma by Targeting TRIB2

**DOI:** 10.1155/2021/9851958

**Published:** 2021-07-26

**Authors:** BioMed Research International

*BioMed Research International* has retracted the article titled “miR-509-5p Inhibits the Proliferation and Invasion of Osteosarcoma by Targeting TRIB2” [1] due to concerns with the reliability of the data. The journal identified that the TRIB2 bands in Figure 4b are duplicated with the p21 bands in Figure 4F when flipped vertically. The authors responded to explain that this was due to an error during the selection of images during the preparation of the manuscript, however, the editorial board determined that the retraction of the article is necessary. The authors agree to the retraction.
